# Bio-Impedance Spectroscopy of Retained Cells Using a Micro-Perforated Sensing Membrane Filtrating Whole Blood Samples under High Flowrate

**DOI:** 10.3390/bios13120996

**Published:** 2023-11-22

**Authors:** Matthieu Sagot, Elise Bou, David Bourrier, Aline Cerf, Hervé Aubert, Christophe Vieu

**Affiliations:** 1 LAAS-CNRS, Université de Toulouse, CNRS, INSA, INPT, 31400 Toulouse, France; 2 SmartCatch, 1 Place Pierre Potier, 31100 Toulouse, France

**Keywords:** blood, filtration, impedance spectroscopy, microfluidics

## Abstract

Blood filtration using micro-fabricated devices is an interdisciplinary topic of research and innovation driven by clinical applications in cytapheresis, cardiovascular disease monitoring, or liquid biopsy. In this paper, we demonstrate that a micro-perforated membrane can be equipped with sensing microelectrodes for detecting, in situ and in real-time, the capture of cellular material during ex vivo filtration of whole blood under high flow rates. This work describes the fabrication process of the sift and detection microdevice. We demonstrate that reliable electrical signals can be measured in whole blood samples flowing inside a fluidic system at typical flow rates, as large as 11.5 mL/min, hence allowing for large-volume sample processing. The in situ monitoring of the electrical impedance of the microelectrodes is shown to characterize the accumulation of living circulating cells retained by the filtrating membrane, opening interesting applications for monitoring blood filtration processes.

## 1. Introduction

Blood is a biological fluid of very high interest for many clinical applications due to the presence of a large variety of disease-linked circulating biomarkers that can be the target of medical diagnosis strategy [1,2,3] but also for removing specific undesirable elements from the circulating blood [4,5]. Blood filtration is, therefore, a process central in hemodialysis [6], cardiovascular disease monitoring [7], and liquid biopsy applications based on the selective capture of Circulating Tumor Cells (CTCs), among other clinical contexts [8].

Clinically, CTCs allow for the detection of asymptomatic early-stage tumors before traditional Computed-Tomography Scan (CT Scan) detection. At another stage of development of the pathology, they can also provide vital insight into minimal residual disease, as well as into the biological mechanisms of tumor progression and drug resistance [9,10,11,12]. Finally, a large interest of the community is turned towards large blood sample processing for both diagnosis and CTC removal with the assistance of hemodialysis or leukapheresis-modified techniques as a potential post-surgery cancer therapy [13,14,15,16].

More generally, whatever the final clinical purpose of blood filtration, different constraints need to be considered for designing the sifting system. Blood is a complex medium containing a huge quantity and variety of cells and proteins exhibiting a viscosity three to eight times greater than water and non-Newtonian behavior when flowing [17]. A large volume of blood needs to be processed either because of the scarcity of the targeted elements (for example, in the case of CTC capture, clinical relevance starts at 5 CTCs/mL of blood) or because the whole circulating blood needs to be expurgated from some adverse entities (such as cell aggregates or circulating microparticles in stroke and cardiovascular diseases [5]). The pore size of the sifting elements needs to be controlled with high accuracy in order to retain only the targeted element driving the biological information in the case of a diagnosis application or driving the pathogenicity in the case of therapeutic applications without impairing the composition of the eluted blood. However, because a large volume of blood is processed and due to the presence of millions of white blood cells (WBC) and billions of red blood cells (RBC) per milliliter of blood, the filters are subjected to clogging due to the unwanted accumulation of material unavoidably retained among time. This drawback appeals to the in situ method capable of sensing the cell density at the surface of these filters during use to monitor their saturation in order to clean their surface or to proceed to their replacement by fresh ones.

In this context, we propose a microfabricated device capable of fulfilling these requirements. The produced sensing devices combine a filtrating membrane with an in situ cellular electrical detection method of the cell density on the filter surface through interdigitated microelectrodes and impedance spectroscopy measurements [18,19,20]. Technological microfabrication brings the ability to control with great accuracy the size of the pores with respect to the size of the targeted elements that require filtration. However, despite using micron-scale filtrating pores and microfabricated devices, we propose a specific design that enables blood filtration at a high flow rate (11.5 mL/min), which is much larger than usual microfluidic devices. Finally, we demonstrate in this contribution that stable electrical measurements can be performed in whole blood at high flow rates in order to monitor the saturation of the filter by retained cells in situ. Our clean room fabricated devices follow robust mass manufacturing processes capable of providing broad access to high-end technology at a relatively low cost per device. Traditionally used to manufacture semiconductors, these technological platforms can provide medical-grade equipment, especially in the field of liquid biopsies, where microfluidics and sensing are performed at the cellular scale.

## 2. Materials and Methods

### 2.1. Manufacturing Process

**Figure 1:**
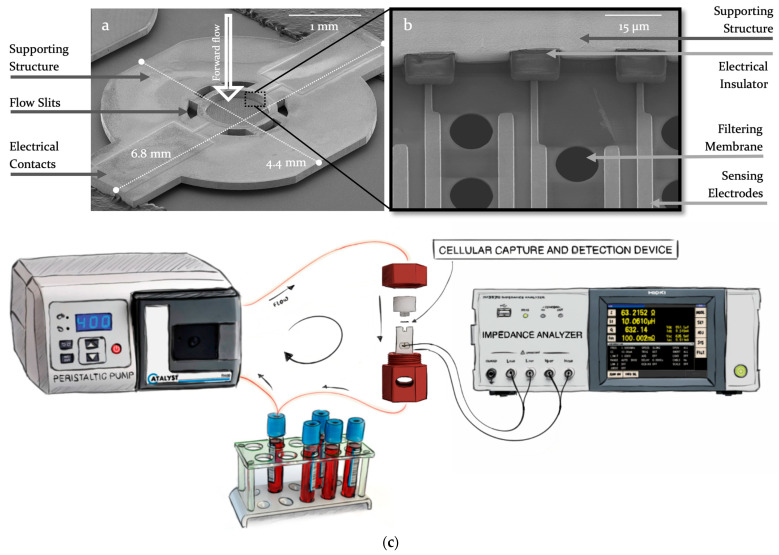
(**a**,**b**) Scanning Electron Micrographs of the Cellular Capture and Detection Device. The whole sensing device is shown in (**a**); (**b**) is a close-up on the filtering membrane in the central part of the device; (**c**) is the experimental setup using a peristaltic pump to flow samples through the Cellular Capture and Detection Device. The microfabricated device is encapsulated in the fluidic system using a 3D-printed holder and connected through spring loaded pins tothe impedance analyzer.

The Cellular Capture and Detection microdevice with integrated electrodes (see Figure 1a,b) is manufactured through 4 main technological steps. The first step consists of the fabrication of a perforated 1.4 µm thick silicon oxide and silicon nitride bilayer (SiO_2_/Si_3_N_4_). This step is followed by the vacuum deposition of 800 nm of gold (Au) and allows the shaping of the electrodes through a lift-off process. A 5 µm layer of dry film (DF) is then deposited to insulate the conductive tracks. Next, an electrolytic growth (Yamamoto-MS A52 Silicon Wafer Plating) of 150 µm of nickel (Ni) forms the part supporting the filtering membrane and allows its handling by conferring an important mechanical resistance. Finally, wet etching of the silicon wafer releases the devices from the silicon substrate and provides the final micro-perforated sensing membrane. All the required patterns obtained at different technological steps are achieved from conventional UV contact photolithography. The manufacturing process is very well suited for mass production as it applies to mature and well-controlled processes of the semiconductor industry. The complete process description is reported in the Supplementary Information (Figure S1).

### 2.2. Design Parameters

The shape of the microdevice is given by the Nickel structure that supports the filtering membrane. The structure is 150 µm thick and has an imprint of 4.4 mm by 6.8 mm to optimize the number of devices on each wafer. It exhibits circular 200 µm wide fluidic slits surrounding the filtering membrane. These slits allow for a partial filtration of the sample: the flow is distributed through both the surrounding slits and the microperforated membrane, allowing releasing the pressure of the filtering membrane and, therefore, achieving larger flow rate for sample processing while reducing mechanical stress on captured cells. The 1.4 µm thick filtering membrane is centered within the nickel supporting structure and covers a circular surface of 570 µm radius, displaying 2000 circular pores of 10 µm diameter each, following a staggered distribution with 8 µm and 16 µm spacing, respectively, vertically and horizontally in between each pore. The electrodes on these devices are interdigitated (IDE) (see supplementary Figure S2) and are composed of two main electrodes with different numbers of parallel fingers. Different electrode designs were considered. Hence, depending on the device, the electrode fingers surround either 60 pores, 1000 pores, or 2000 pores of the membrane, thus providing, respectively, 2, 34, and 86 fingers, also called single coverage, partial coverage, or full coverage of the microperforated membrane. The length of each finger varies since the membrane is circular; the width (4 µm) is kept constant. Each electrode is connected through a 2 mm long track to a 900 µm by 680 µm contact pad, enabling electrical contact and characterization with external equipment.

### 2.3. Experimental Setup

As depicted in Figure 1c, the experimental setup consists of a fluidic system to transport the different solutions through the filter, an electrical system for measuring the impedance of the device, and a holder that encapsulates this device within the fluidic channel and relocates electrical contacts outside of the holder. The fluidic setup is composed of a peristaltic pump (Catalyst FH100) and a 1.6 mm internal diameter silicone biocompatible tubing (Masterflex™ 06424-14). The electrical setup consists of an impedance analyzer (HIOKI IM3570) connected through shielded coaxial wires to the holder pins.

The two inner pieces of the holder, machined from a biocompatible material (POMc, polyoxymethylene), can be intertwined in order to hold the microdevice in place and close the fluidic channel. Two toroidal gaskets are used above and below the microdevice to seal it in the fluidic canal. Top and bottom sides of the holder present threaded slots for inlet and outlet fluidic connectors. Spring-loaded pogo pins (Harwin P70-2300045R) are inserted to contact the pads leading to the electrodes on the microdevice. A second 3D printed (FDM, Fused Deposition Modeling) layer encapsulates the first two pieces to hold them intertwined using a screw mechanism. Slits and openings ensure the accessibility of both the electrical and fluidic connectors are accessible when closed.

The solutions used during experiments include ethanol, PBS (Gibco 1X), RPMI (Gibco 1640), Fetal Bovine Serum (FBS), and paraformaldehyde (formalin, neutral buffered 10%). The blood samples are purchased from Etablissement Français du Sang (EFS, 6 mL whole blood tubes with EDTA coating). Bleach and deionized water are used to clean and sanitize the holder.

### 2.4. Calibration and Control Protocol

The impedance analyzer calibration is performed before each experiment using two specifically designed devices for both the open and short circuit calibration, which are inserted in the holder, thus compensating for length of cable and potential parasitic effects at the junctions of the spring-loaded pins. These devices were fabricated following the same fabrication process as the operational ones but were provided with specific electrode designs that are shown in supplementary Figure S3). The calibration protocol is validated through control experiments within PBS solutions following the experimental protocol described in the next section, where signal stability over time is evaluated.

### 2.5. Experimental Protocol

The microdevice is inserted in the holder, which is connected both to the fluidic system and impedance analyzer. Priming steps include flowing 15 mL of ethanol to improve the wettability of the device and remove any trapped air bubbles. Next, 15 mL of PBS is used to replace ethanol in the system and establish physiological conditions. These priming steps are applied in an open-loop setup and in reverse flow to remove eventual impurities. The reference impedance spectrum is measured in PBS. The blood sample of 12 mL is then processed in a closed loop for 10 min in forward flow at 11.5 mL/min. During this time, impedance spectroscopy measurements from 1 kHz to 5 MHz at 50 mV are performed every 2 min until the end of the process. Post-processing steps include the rinsing of the fluidic system with 20 mL of PBS in forward flow, followed by the introduction of paraformaldehyde for cell fixation. The incubation takes 20 min. The fluidic system is then washed off paraformaldehyde with another 20 mL of PBS before retrieving the holder and the microdevice from the setup for optical observation. The blood sample can be changed by PBS, cell culture media (RPMI 5% FBS), or blood plasma for control experiments.

## 3. Results

### 3.1. Impedance Evolution

**Figure 2:**
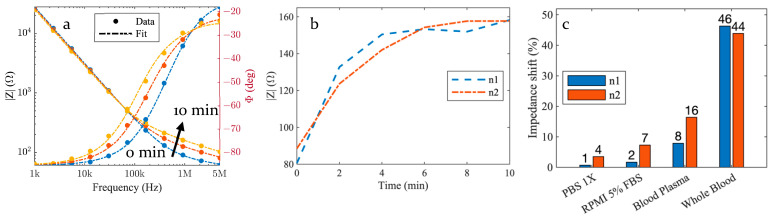
(**a**) Six impedance spectra are measured every 2 min for 10 min (at 0, 2, 4, 6, 8, and 10 min) along the filtration process of a blood sample through the Cellular Capture and Detection Device and three of them are shown in the figure as the impedance modulus (**left**) and phase (**right**) at 0 min, 2 min and 10 min (respectively, blue, red, and yellow). The dashed curves are the results of a fitting based on the equivalent electrical circuit model detailed in Section 4 (**b**). Same measurement data as in panel (**a**). plotting the time evolution of the measured impedance at 1 MHz for two identical independent experiments (**c**). Comparison of the impedance relative shift at 1 MHz over 10 min of sample processing between saline solution PBS 1X, cell culture medium (RPMI 5% FBS), blood plasma control samples, and whole blood sample.

Figure 2a exhibits the typical impedance spectrum profile and gives the magnitude and phase of the device impedance as a function of the frequency from 1 kHz to 5 MHz. Plotted data were obtained at 0 min, 2 min, and 10 min under whole blood circulation through the device. Between 1 kHz and 100 kHz, the device impedance does not change significantly when the blood flows, while in the 100 kHz to 5 MHz range, the impedance magnitude $\left|Z\right|$ increases gradually over time. Measured impedance spectra can be fitted using a very simple electrical circuit, as discussed in the upcoming sections. In order to better visualize the time variation of the impedance, the magnitude $\left|Z\right|$ at 1 MHz is displayed in Figure 2b as a function of time t for two identical experiments and nominally identical devices. We can notice an increase in the magnitude during the first 4 min and a plateau, indicating a saturation-like behavior before the end of the experiment.

### 3.2. Control Samples

In Figure 2c, we report the impedance shift between whole blood circulation and three control experiments, where a saline solution (PBS 1X), a cell culture medium (RPMI with 5% FBS), and blood plasma are used. The impedance relative shift S (in %) is derived from measurement results obtained at 1 MHz, as follows:

(1) $$S=100\times \frac{{\left|Z\right|}_{10\;min}-{\left|Z\right|}_{0\;min}}{{\left|Z\right|}_{0\;min}}$$

where ${\left|Z\right|}_{t}$ denotes the measured magnitude of the impedance at time t [minutes]. The relative shift during the 10 min experiments with whole blood ranges from 44% to 46%, while it is limited to 8% to 16% for blood plasma, 2% to 7%, and 1% to 4% for the culture medium and the PBS, respectively.

## 4. Discussion

### 4.1. Electrical Circuit Model

We propose herein an equivalent electrical circuit with the aim to model accurately the microdevice electrical behavior by setting a baseline using control experiments. This baseline will then be used to physically interpret the observed shifts of impedance spectroscopy data. The model considers the electrochemical effects through the faradaic impedance and the electrical properties of the medium.

### 4.2. Interface Impedance Model

In this electrical circuit,${\;C}_{dl}$ models the ionic double-layer capacitor at the interface between the electrode and the electrolyte [21,22]. This capacitive behavior is observable as a downward slope in the impedance modulus spectra together with a −90° phase between 1 kHz and 100 kHz, where it dominates the measured impedance values. In our case, a Constant Phase Element (CPE) element, first introduced by Kenneth S. Cole [23], is used to model this double-layer capacitance as a non-ideal capacitor of impedance general form described as follows:

(2) $$Z_{CPE}=\frac{1}{Q_{0}{\left(i\omega \right)}^{n}}$$

with $Q_{0}[S.s^{n}]$, $n\in \left[0,1\right]$ denotes the so-called ideality coefficient and $\omega \;[rad.s^{-1}]$ designates the angular frequency.

### 4.3. Medium Impedance Model and Dispersion

Modeling the impedance modulus plateau and its phase tending towards a resistive behavior (0°) in the frequency region of 100 kHz to 5 MHz, together with its evolution along filtration, requires taking into consideration the electrically dispersive nature of the media. Indeed, the dielectric parameters of blood are dispersive; that is, they depend on the frequency [24,25]. K. R. Foster and H. P. Schwan reviewed and reported in 1989 the complex admittance $Y^{*}$ of a dispersive medium in between an ideal parallel plate capacitor using a parallel RC equivalent circuit [26]:

(3) $$Y^{*}=G+j\omega C\;=(A/d)(\sigma +j\omega \epsilon_{0}\epsilon_{r})$$

where $\epsilon_{0}$ is the vacuum permittivity, and $\epsilon_{r}$ and σ are, respectively, the relative permittivity and the conductivity of the inter-plate medium. However, it is common for coplanar capacitors to use a cell constant κ in order to account for the geometrical parameters of this device. Therefore, the whole structure can be modeled through a single variable κ, which is described as follows for interdigitated sensors [27]:

(4) $$\kappa =\frac{2}{\left(N-1\right)L}\;\frac{K(k)}{K(\sqrt{1-k^{2}})\;}\;[m^{-1}]$$

where K(k) is the complete elliptic integral of the first kind defined as follows:

(5) $$K(k)=\int_{0}^{1}\frac{dt}{\sqrt{\left(1-t^{2})(1-k^{2}t^{2}\right)}}$$

with $k=cos(\nu \frac{\pi }{2})$ where $\nu =\frac{w}{s+w}$. The variable κ depends on the number of digits *N*, their length *L*, and, through the metalization ratio ν, on their width W and space S between the fingers of the electrode. Thus, the impedance of the medium is given as follows:

(6) $$Z_{m}=\frac{1}{Y^{*}}=\frac{G-j\omega C}{G^{2}+{\left(\omega C\right)}^{2}}=\kappa \frac{\sigma (\omega )-j\omega \epsilon_{0}\epsilon_{r}(\omega )}{{\sigma (\omega )}^{2}+{\left(\omega \epsilon_{0}\epsilon_{r}(\omega )\right)}^{2}}\;$$

K. R. Foster and H. P. Schwan reported in [26] the relaxation theory by which the frequency dependence of the dielectric parameters is accounted for through the Debye equation [28] for a single relaxation region:

(7) $$\epsilon^{*}=\epsilon_{\infty }+\frac{\epsilon_{s}-\epsilon_{\infty }}{1+j\omega \tau }$$

where $\epsilon_{\infty }$ is the permittivity at high frequencies (ωτ≫1), $\epsilon_{s}$ denotes the permittivity at low frequencies (ωτ≪1) and τ [s] the dielectric relaxation time. The magnitude Δϵ of the dispersion is then given by $\epsilon_{s}-\epsilon_{\infty }$. The Cole–Cole equation [29] introduces the distribution parameter α in order to empirically account for the broadening of dispersion regions of complex biological materials. W. D. Hurt modeled in 1985 the dielectric spectrum by adding the static ionic conductivity term $\sigma_{i}$ into the summation of n Cole–Cole dispersion [30], as follows:

(8) $$\epsilon^{*}\left(\omega \right)=\epsilon_{\infty }+\sum_{n}\frac{\Delta \epsilon }{1+{\left(j\omega \tau_{n}\right)}^{1-\alpha_{n}}}+\frac{\sigma_{i}}{j\omega \epsilon_{0}}$$

Parameters used to predict the dispersion of blood with the above-mentioned equation were reported by S. Gabriel et al. in 1996 and are the result of a fitting process over experimental data [24].

We propose herein to model the global impedance of the micro-device from the series combination of the interface impedance $Z_{i}$ and of the medium impedance $Z_{m}$:

(9) $$Z=Z_{i}+Z_{m}=\frac{1}{Q_{0}{\left(i\omega \right)}^{n}}+\kappa \frac{\sigma (\omega )-j\omega \epsilon_{0}\epsilon_{r}(\omega )}{{\sigma (\omega )}^{2}+{\left(\omega \epsilon_{0}\epsilon_{r}(\omega )\right)}^{2}}$$

where $\epsilon_{r}(\omega )$ denotes the real part of $\epsilon^{*}\left(\omega \right)$, while $\sigma (\omega )/\epsilon_{0}\omega$ is given by the imaginary part of $\epsilon^{*}\left(\omega \right)$. Fitting results obtained from this model combining the impedance of the coplanar sensor together with the electrical dispersion of the medium (respectively, Equations (8) and (9) are in very good agreement with measurement data for PBS, culture medium, and whole blood. Supplementary Figure S4 shows the variation of the interface and medium impedance as a function of time for culture medium and whole blood together with the fitting values of the electrical model. We observe good stability over the experiment for the interface impedance $Z_{i}$ that models the double layer effects, with 4% to 12.7% deviation in culture medium and whole blood, respectively. On the other hand, the medium impedance $Z_{m}$ shifted by 100% in whole blood and by 7.4% in culture medium.

### 4.4. Impedance Spectra over Time

The stability of the impedance spectrum at low frequencies (between 1 kHz and 100 kHz; see Figure 2a) originates from the predominance of the ionic double-layer capacitance. As it is unchanged under blood circulation, the low-frequency impedance spectrum cannot be used to detect any adsorption or cell capture on the micro-perforated membrane. By extension, the impedance stability also indicates that variations due to environmental factors (including temperature fluctuations) are negligible and that microelectrodes are not damaged during experiments. Above the cut-off frequency of 100 kHz, the measured impedance provides information about the dielectric properties of the region close to the gaps between the interdigitated microelectrodes through the electrical circuit model. The impedance variation over 10 min experiments correlates with the capture of white blood cells on the micro-perforated membrane. Indeed, the device impedance with pure PBS, cell culture medium (RPMI 5% FBS), and blood plasma exhibits comparatively much smaller variations. Consequently, the time variation of the impedance observed in the blood is not dominated by the electrolyte itself or by the deposition of proteins. Instead, it originates from the capture of blood cells. WBC capture is actually confirmed by optical inspection of the device after 10 min of blood circulation using nucleus staining with a Hoechst label. Supplementary Figure S5 exhibits a large number of nucleated blood cells captured by the micro-perforated membrane. The equivalent electrical circuit model allows for an accurate description of the impedance spectra, and its intrinsic component values evolution supports the detection through an increase in resistivity in between the sensing electrodes. It is also interesting to note that, due to the formation of an RC equivalent circuit, the induced cut-off frequency, which evolution can be seen on the phase diagram, is tightly linked to the resistive component, thus providing another way of electrical detection based on the measurement of cut-off frequency shifts which may become useful for further studies.

### 4.5. Capture and Detection Kinetics

The increase in impedance magnitude $\left|Z\right|$ of the filtering microdevice at 1 MHz during the first 4 min of the experiments (Figure 2b) reveals the capture of WBC by the micro-perforated membrane. After a 4 min capture phase, a plateau is reached. This plateau can be explained either by the decrease in the electrical sensitivity of the device under cell accumulation and/or a decrease in the capture efficiency of this device. Indeed, as the number of captured cells increases, the sensitivity is expected to decrease if the contribution to the global impedance of the next captured cell is lower than that of the previous one. However, electrical simulations performed using *COMSOL Multiphysics* and cell deposition in between the electrodes of our device did not reveal such an effect. We, thus, conclude that the plateau correlates to the limit at which the filtering membrane becomes saturated. Supplementary Figure S6 shows the capture kinetics evaluated using optical microscopy on a micro-perforated membrane without integrated microelectrodes. In this experiment, PC9 cells were spiked in a culture medium. The number of cells observed on the micro-perforated membrane was recorded in situ by optical inspection. The obtained results exhibit the same saturation trend between 4 and 10 min. We, thus, derive that the integrated microelectrodes can detect and measure in real time the rate of cell capture taking place on the micro-perforated membrane in unprocessed whole blood.

## 5. Conclusions

In this paper, we proposed a new device for ex vivo blood sample filtration, combining a filtrating membrane with an in situ cellular electrical detection method of the cell density on the filter surface through interdigitated microelectrodes. The produced devices turned out to allow both the cell capture and electrical detection of the cell density at the filter surface at a high flow rate (11.5 mL/min). The cleanroom fabrication process of these devices is described, and the pore size of the sifting elements can be controlled with high accuracy and adapted to the clinical context of use in order to retain only the targeted element driving the biological information. Together with the fabrication and electrical implementation, we developed an equivalent electrical circuit, which accurately models the complexity of the blood sample and allows analyzing the capture kinetics.

Previous studies published in the field of blood filtration focus on the complete recovery of the targeted biomarker with the aim of increasing the throughput by parallelizing devices. However, the scalability of these systems is questionable, as the size of the microfluidic systems is often tightly correlated to their capture efficiency [31,32]. Our approach to filtration significantly differs from this concept by focusing on a high-flow rate device combined with the use of electrical cell detection that does not require any extensive parallelization, thus alleviating scalability issues.

Finally, further developments on the sensing electrodes could enhance the frequency bandwidth of the sensor, thus giving access to the specific dielectric dispersion of collected biological samples. Identifying the elements retained by the filter through their bio-electrical signature would be a key step for real-time, in situ, and label-free analysis of blood circulating biomarkers.

## Data Availability

The data presented in this study are available on request from the corresponding author. The data are not publicly available.

## Supplementary Materials

The following supporting information can be downloaded at: https://www.mdpi.com/article/10.3390/bios13120996/s1, Figure S1: Fabrication Process of the Capture and Detection Device; Figure S2: Design of the interdigitated electrodes; Figure S3: Calibration devices; Figure S4: Fitting result of the electrical equivalent circuit model; Figure S5: Capture and detection device optical inspection; Figure S6: Cell capture dynamics.
