# Supplementary material for: Bio-Impedance Spectroscopy of Retained Cells Using a Micro-Perforated Sensing Membrane Filtrating Whole Blood Samples under High Flowrate
Source: Biosensors (Basel). 2023 Nov 22;13(12):996. doi: 10.3390/bios13120996 (PMC10741909; doi:10.3390/bios13120996)
Supplement: Supplementary file 1 [file biosensors-13-00996-s001.zip › biosensors-2674382-supplementary.pdf]

Supplementary Information

Supplementary Figure S1: Fabrication Process of the Capture and Detection Device

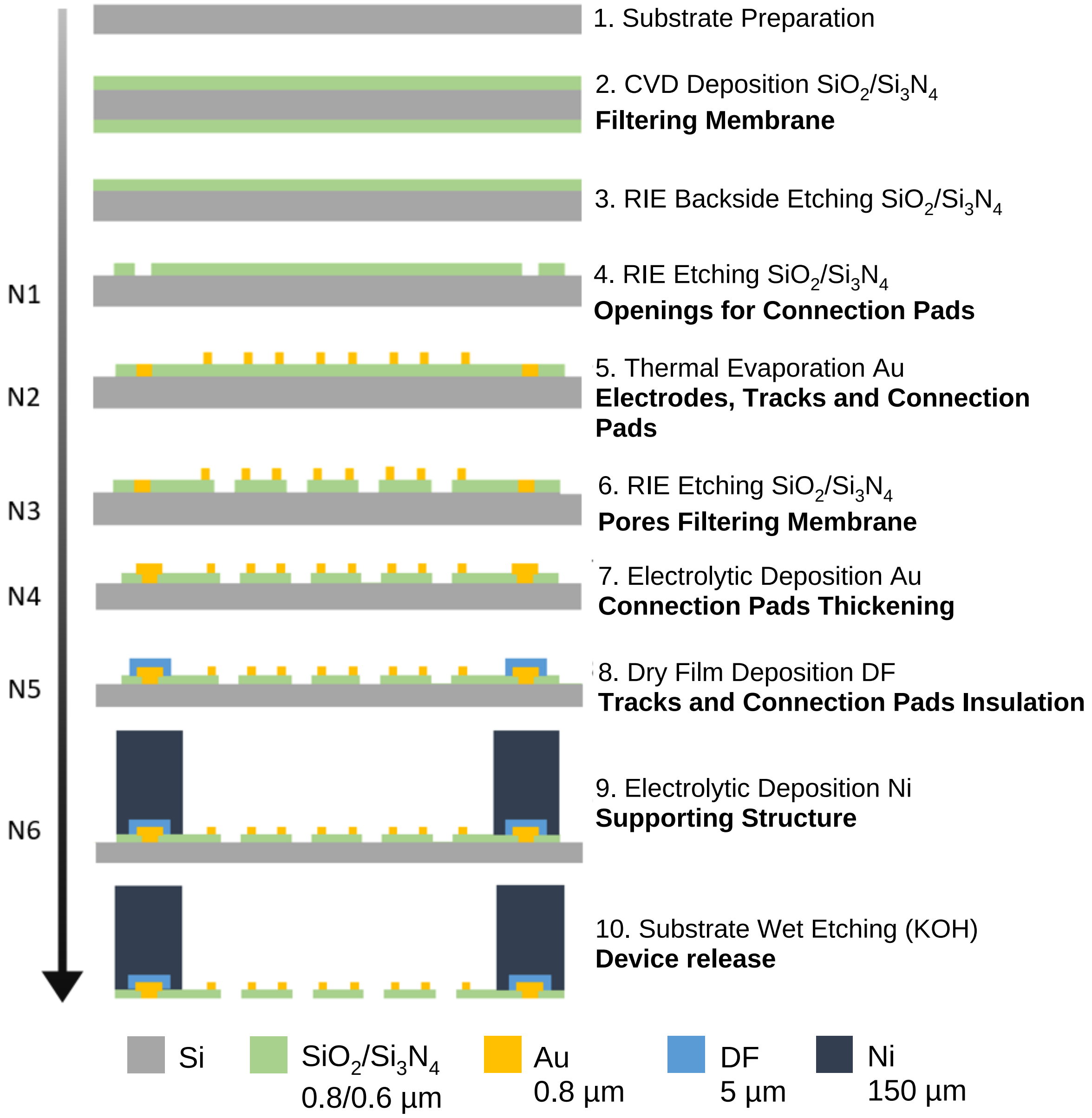

The fabrication process is described through 10 main steps. It includes six masks (N1 to N6) with six UV photolithography steps.

1. Substrate preparation. A 4-inch high-resistivity silicon wafer is cleaned in a piranha solution (50% H_2SO_4 + 50% H_2O_2). Deoxidation is then performed in a 5% HF solution.
2. Chemical vapor deposition. Thermal oxidation is performed on the substrate to obtain a thick SiO_2 layer. A SiNx film is then deposited by LPCVD. These deposits are made on both sides of the wafer at the same time.

3. RIE Backside etching. RIE etching is performed with a CF_4 ICP plasma on the backside of the wafer to remove the SiO_2/SiN_x bilayer. The gas pressure is 40 cm3/min, the RF power is 40W and the etching time is 13 minutes.
4. Opening of the connection pads (N1). An O_2 plasma process (time: 5 minutes, power: 800 W, flow rate: 400 cm3/min) is carried out on the wafer in order to eliminate possible organic residues. A hexamethyldisilazane (HMDS) deposit is performed on the wafer to ensure the adhesion of the resist on the oxidized surface. A positive resist, ECI, is spin-coated in two steps. A first deposition (speed: 5000 rpm/min, acceleration: 4000 rpm/min2, time: 30 seconds) with annealing at 90°C for 30 seconds followed by a second deposition (speed: 1000 rpm/min, acceleration: 4000 rpm/min2, time: 30 seconds) and annealing for 1 minute 15 seconds to achieve a total layer thickness of 2.4 µm are carried out. Trimming of the resist is performed with acetone. UV exposure is performed with a dose of 250 mJ/cm2 in a contact vacuum, followed by a 1 min 15 sec post-exposure bake at 110°C. After resist development, RIE etching is performed in an ICP reactor with CF_4 plasma (pressure: 40 cm3/min, power: 40W, time: 10 min). Using the resist pattern as an etching mask, the removal of the resist is performed in an acetone bath for 2h and then in a DMSO bath heated at 80°C for 1h. An O_2 plasma process (time: 5 minutes, power: 800 W, flow rate: 1000 cm3/min) is carried out to remove possible resist and solvent residues.
5. Definition of microelectrodes, connection tracks and contact pads (N2). An O_2 plasma process (time: 5 minutes, power: 800 W, flow rate: 400 cm3/min) is performed, followed by HMDS deposition. A negative resist, NLOF, is spin-coated (speed: 5000 rpm/min, acceleration: 4000 rpm/min2, time: 30 seconds) to obtain, after annealing at 105°C for 1 minute, a layer thickness of 2.5 µm. UV exposure is performed with a dose of 250 mJ/cm2 in vacuum contact, followed by a 1 min post-exposure cure at 110°C. After resist development, a metal layer is deposited by thermal evaporation, which consists of a bilayer of chromium and gold, with the chromium providing adhesion to the substrate. Lift-off is performed in a DMSO bath for about two hours, and the wafer is then rinsed with acetone and deionized water.
6. Opening the pores of the filter membrane (N3). This step is identical to the one described in point 4 for UV photolithography and the etching process, except that the duration of the etching is 12 min.
7. Formation of the connection vias (N4). A seed layer consisting of a bilayer of titanium and copper is deposited by thermal evaporation on the wafer. An O_2 plasma process (time: 2 minutes, power: 200 W, flow rate: 400 cm3/min) is performed on the wafer. A negative resist, AZ15NXT, is coated using a spinning machine (speed: 5000 rpm/min, acceleration: 4000 rpm/min2, time: 30 seconds). Annealing is conducted at 105°C by positioning the wafer for 30 sec at 5 mm, 30 sec at 1 mm from the plate, then 30 sec in contact and finally 1 min 30 in vacuum contact. The resulting resist thickness is 19 µm. UV exposure is performed with a dose of 1600 mJ/cm2 in soft contact. Post-exposure curing is performed at 110°C with the wafer positioned for 30 sec at 5 mm, 30 sec at 1 mm from the plate, then 30 sec in contact and finally 1 min 30 in vacuum contact. After the development of the resist, an O_2 plasma process is performed on the wafer (time: 2 minutes, power: 200 W, flow rate: 400 cm3/min) to remove all resist leftovers. Next, 1 µm gold electroplating is performed. The resist is removed with acetone, followed by an NF52 bath at 80°C for 5 min and an O_2/CF_4 plasma process (time: 2 min, power: 200 W, flow rates: 200 cm3/min for O_2 and 40 cm3/min for CF_4).

The copper of the seed layer is etched with a piranha solution (1% H_2SO_4 + 1% H_2O_2+98% H_2O) and the titanium is etched with 5% HF buffer, then the wafer is rinsed with DI water.
8. Insulation of the contacts and connection tracks (N5). An O_2 plasma process (time: 2 minutes, power: 200 W, flow rate: 400 cm3/min) is performed on the wafer. A dry film (DF-1000, 5 µm thick) is deposited with a Nanonex press, equipment conventionally used for the nanoimprint technique (pumping time: 2 min, preprint: 30 °C 15 psi, print 65 °C 30 psi, time: 2 min, 55 °C). The UV exposure is performed with a dose of 550 mJ/cm2 in hard contact. Post-exposure baking is performed at 100°C for 3 minutes. The film is developed in a cyclohexanone bath for 4 min. The wafer is then rinsed with isopropanol. An O_2 plasma (time: 2 minutes, power: 200 W, flow rate: 400 cm3/min) proves is performed in order to remove the resist and solvent residues on the substrate. Hard bake annealing is performed (60°C for 4 min, 80°C for 2 min, 100°C for 2 min, 120°C for 13 min). An O_2 plasma process (time: 2 min, power: 200 W, flow rate: 400 cm3/min) is again performed to remove any persistent residues.
9. Formation of the support part (N6). A seed layer consisting of a bilayer of titanium and copper is sputtered onto the wafer to obtain a conformal deposit on the structured surface. Two dry WBR-2100 films, 100 µm thick, are laminated to the wafer at 50°C (pressure: 2.5 bar, speed < 0.5 m/s). Annealing is performed at 65°C for 10 min. UV exposure is performed with the MA150 mask aligner with a dose of 1100 mJ/cm2 in vacuum contact. Post-exposure baking is performed at 105°C for 2 min. The development is carried out in a sodium carbonate solution NaCo3 with agitation for about 15 min. After development of the resist, a CF_4/O_2 plasma process (time: 2 min, power: 200 W, flow rate: 400 cm3/min) is performed on the wafer. Electroplating of nickel is performed. The resist is removed with acetone, followed by a NF52 bath at 80°C for 45 min. The seed layer is etched as described in step 7, corresponding to the N4 level and the electrochemistry step of the connecting vias.
10. Etching of the silicon substrate and release of the devices. The silicon is etched with KOH. The released microdevices are then rinsed in a DI water bath. The etching of the seed layer is carried out piece by piece. Copper is etched with a piranha solution (1% H_2SO_4 + 1% H_2O_2+98% H_2O) and titanium is etched with a 5% HF buffer. The microdevices are then rinsed in a DI water bath without agitation.

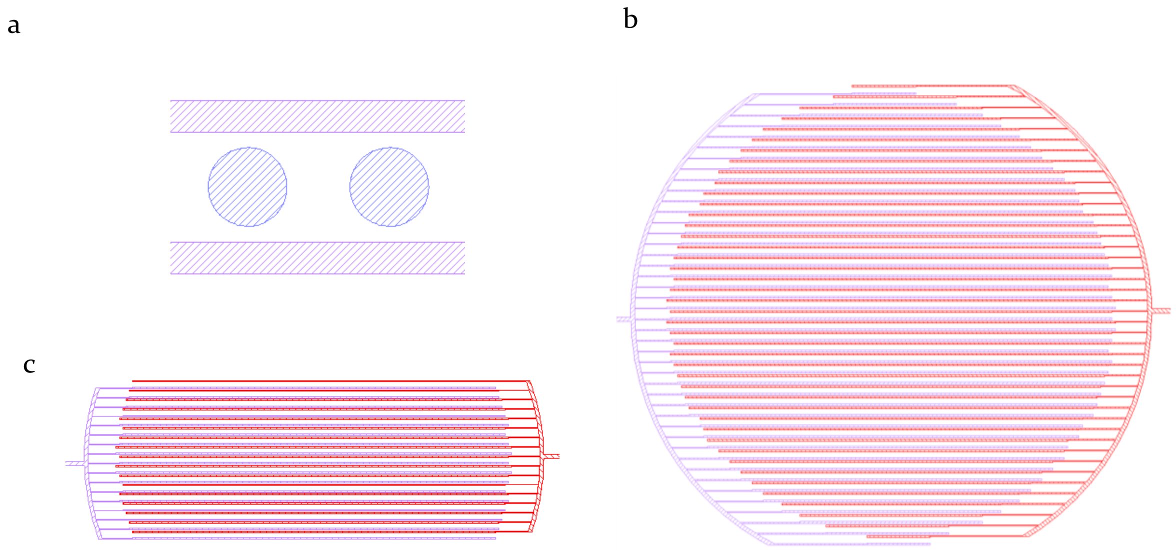

Supplementary Figure S2: Design of the interdigitated electrodes with 2 pores in between the sensing electrodes (a). This design was extended to the full coverage (b) and partial coverage (c) of the membrane. Access lines and contact pads are not represented here.

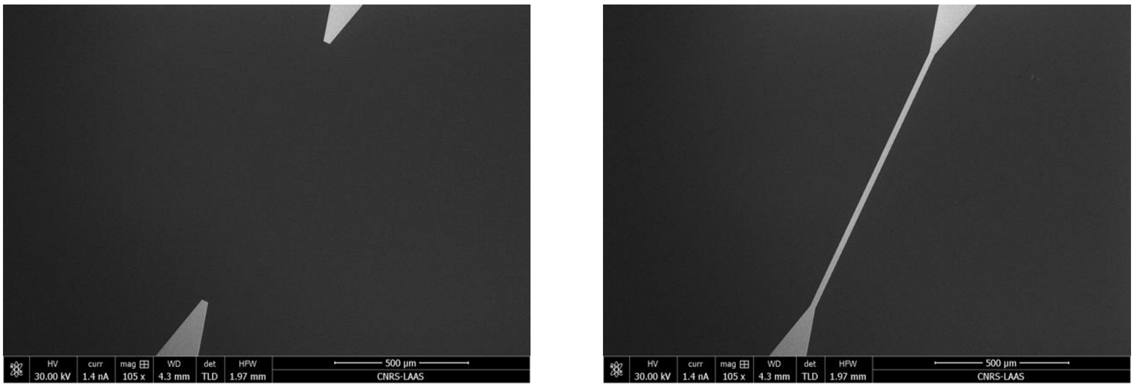

Supplementary Figure S3: SEM Micrographs showing electrode designs of the calibration devices: open circuit calibration (left) and short circuit calibration (right).

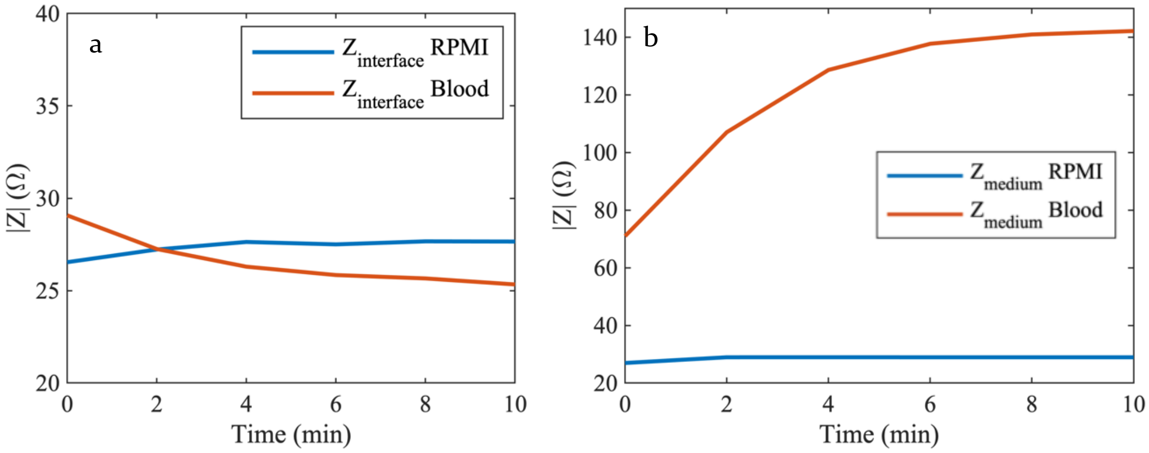

c	Q_0 $[S.s^n]$	n	κ $[m^{-1}]$	σ_i $[S.m^{-1}]$	$\Delta\epsilon_r$
0 min	$8{,}86.10^{-9}$	0.9505	50	0.63	56; 5337
2 min	$9.32.10^{-9}$	0.9489	50	0.35	56; 6973
10 min	$9.87.10^{-9}$	0.9489	50	0.24	56; 6437
with the constants: α = [0.32; 0.32]; τ = [$8.38.10^{-12}$; 441.10^{-9}]; ϵ_0= $8.854.10^{-12}$; ϵ_∞= 4.					

Supplementary Figure S4: Fitting result of the electrical equivalent circuit model for impedance evolution in whole blood and in culture media (RPMI 5% SVF) at 1MHz over 10 minutes of experiment. **a** Plot of the interface impedance evolution. **b** The medium impedance as described in the section equivalent circuit model. Table **c** reports the fitting values of the electrical model for three impedance spectra in whole blood.

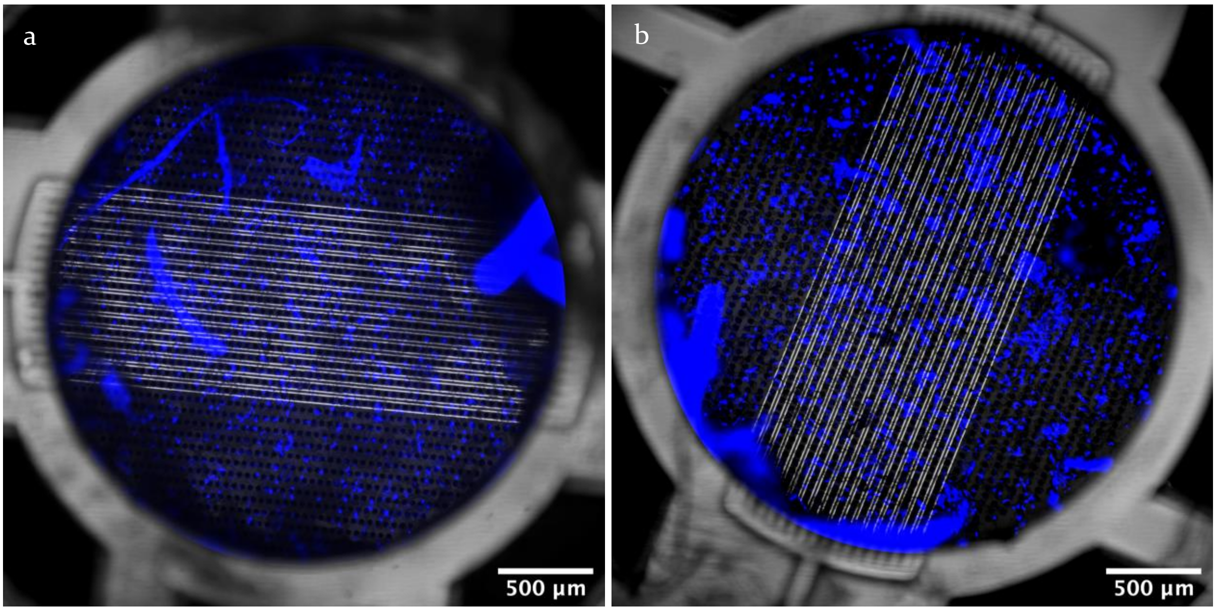

Supplementary Figure S5: Overlay of bright field and colored Hoechst nucleus staining images with 10x objective. Both images show the capture and detection device after 10 min of whole blood processing on two identical devices.

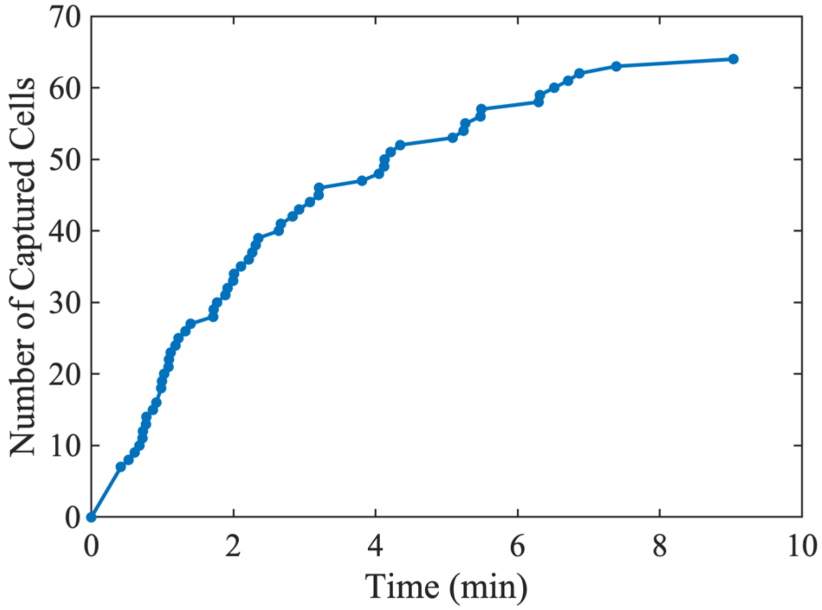

Supplementary Figure S6: Cell capture dynamics obtained using a previous microdevice without integrated electrodes exhibiting a membrane of 137 pores.
